# Recent advances in data- and knowledge-driven approaches to explore primary microbial metabolism

**DOI:** 10.1016/j.cbpa.2023.102324

**Published:** 2023-08

**Authors:** Bartosz Jan Bartmanski, Miguel Rocha, Maria Zimmermann-Kogadeeva

**Affiliations:** 1 Genome Biology Unit, European Molecular Biology Laboratory, Heidelberg, Germany; 2 Centre of Biological Engineering, University of Minho, Campus of Gualtar, Braga, Portugal

**Keywords:** Metabolomics, Microbiota, Metabolic networks, Machine learning, Deep neural networks, Genome-scale models, Multi-omics integration

## Abstract

With the rapid progress in metabolomics and sequencing technologies, more data on the metabolome of single microbes and their communities become available, revealing the potential of microorganisms to metabolize a broad range of chemical compounds. The analysis of microbial metabolomics datasets remains challenging since it inherits the technical challenges of metabolomics analysis, such as compound identification and annotation, while harboring challenges in data interpretation, such as distinguishing metabolite sources in mixed samples. This review outlines the recent advances in computational methods to analyze primary microbial metabolism: knowledge-based approaches that take advantage of metabolic and molecular networks and data-driven approaches that employ machine/deep learning algorithms in combination with large-scale datasets. These methods aim at improving metabolite identification and disentangling reciprocal interactions between microbes and metabolites. We also discuss the perspective of combining these approaches and further developments required to advance the investigation of primary metabolism in mixed microbial samples.

## Introduction

Microorganisms are found in virtually every environment on Earth, from hydrothermal vents in the deep ocean and volcanic craters to industrial buildings, soil, plants, animals, and humans [1, 2, 3]. Microbes can greatly shape and affect their environment by producing or consuming chemical compounds with different properties, supplying essential nutrients to their hosts, detoxifying wastewater treatment plants, or affecting drug metabolism in humans [4, 5, 6, 7, 8]. The broad metabolic potential of microorganisms is harnessed in food, biotechnological, and pharmacological industries [9]. Although numerous microbial metabolism products are known to date, this might be only the tip of the iceberg, and it is imperative to understand how various species metabolize different compounds and which products arise from these transformations to assess and harness the effects of microbial metabolism on the health of our planet and its inhabitants.

With the rapid development of metabolomics and sequencing technologies in the past decades, it has become possible to detect hundreds to thousands of small molecules and identify species composition in different microbial environments [10, 11, 12, 13]. However, computational analysis of microbial metabolomics data remains challenging. The first challenges in analyzing raw data are peak-picking, compound identification, and annotation, which are inherent to all metabolomics datasets acquired with mass spectrometry (MS) [14, 15, 16] and nuclear magnetic resonance (NMR), recently reviewed by Judge and Ebbels [17]. Various approaches exist for compound identification based on their accurate mass, retention time, and fragmentation pattern, usually through comparison to databases, yet a large percentage of the detected compounds often remains unknown [18]. After compound identification and annotation, a specific challenge that arises in microbial metabolomics is separating the sources of metabolites in mixed samples, which usually contain multiple species, whose identity is often assessed by sequencing [15,19]. One of the main obstacles is the unknown metabolic potential of single microbes, since many microorganisms are difficult or impossible to culture in laboratory conditions, and their genomes remain poorly annotated [3,10,11]. Large-scale experimental datasets characterizing the metabolic potential of single microbial species *in vitro* have just started to become available [20], providing essential information to improve interpretation and separation of microbial metabolic activity in mixed samples.

**Figure 1:**
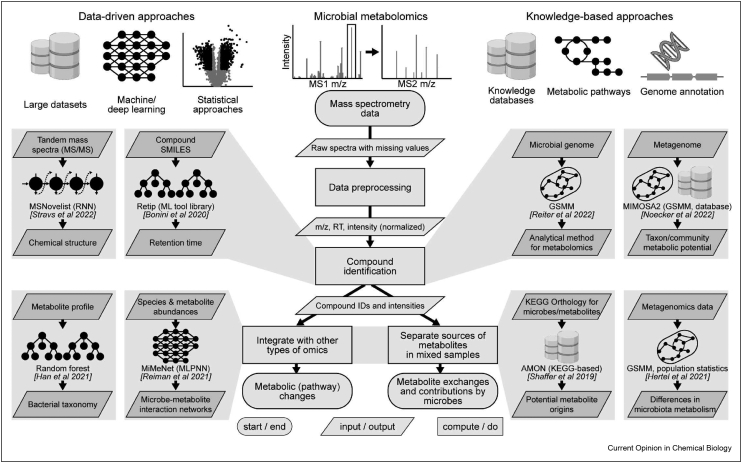
Schematic diagram of the steps in metabolomics data analysis, starting from the raw mass spectrometry data, all the way to the integration with other omics datasets and separating sources of metabolites in mixed samples. Both data-driven (left) and knowledge-based (right) approaches can be used at each step of the analysis pipeline. Selected studies are schematically depicted with the corresponding inputs and outputs. GSMM - genome-scale metabolic model; KEGG - Kyoto Encyclopedia of Genes and Genomes database; *m*/*z* - mass over charge ratio; ML - machine learning; MLPNN - multi-layer perceptron neural network; MS - mass spectrum; RNN - recurrent neural network; RT - retention time.

In this short review, we focus on the most recent advances in the computational analysis of primary microbial metabolomics data: data-driven and knowledge-based approaches (Figure 1). More in-depth reviews of the modern computational methods in metabolite identification were recently published by Blaženović et al. [18] and Nguyen et al. [21], software and tools were summarized by Misra [22], machine and deep learning applications in metabolomics were reviewed by Liebal et al. [16], Sen et al. [23], Antonakoudis et al. [24], Pomyen et al. [25], and Mendez et al. [26], while secondary metabolism and its computational analysis were recently reviewed by Atanasov et al. [27] and Blin et al. [28]. Data-driven approaches are dominated by the machine learning field, and especially deep learning, which has seen an explosion of interest in recent years, owing to decreased computational cost, algorithmic advances, and ever-growing amounts of data. In metabolomics, it is used in data preprocessing, metabolite annotation, and various post-processing steps, such as integration with other types of omics datasets. Knowledge-based approaches rely on database information about metabolites, their properties and potential sources. To analyze and interpret microbial metabolomics data, they often make use of genome-scale metabolic models (GSMMs) and networks, which represent the current knowledge about the biochemical reactions inside the cells reconstructed based on microbial genome annotation [29]. Such models can facilitate metabolite annotation, integration with other omics datasets, and identification of metabolite sources in mixed samples since they define metabolites that can be consumed and produced by a given organism (Figure 1). While both data-driven and knowledge-based approaches are continuously undergoing rapid computational developments, both approaches would benefit from more experimental data on metabolic potential of single microbes and identification of novel compounds that microbes can produce or consume.

## Data-driven approaches: machine and deep learning

Due to their overwhelming effectiveness across a wide range of applications, machine learning and specifically deep learning methods are becoming increasingly popular. In computational metabolomics, machine and deep learning methods have been applied across most analysis steps, from data preprocessing, compound identification and quantification to integration with other omics datasets, reviewed in-depth by Sen et al. [23]. In contrast to other data types, the often small sample sizes and heterogeneous nature of metabolomics data require new types of deep neural networks (NN) to benefit computational metabolomics [23]. As for any machine learning task, a crucial step for the success of NNs is data preprocessing and normalization. There are various ways to normalize the data, which can impact the training of the NN and, as a result, the conclusions reached. Abram and McCloskey have recently reviewed various preprocessing steps of metabolomics data and determined that logarithm of the fold change is the best normalization method when the performance of the NNs was assessed based on sample classification or joint metabolite reconstruction tasks for a set of prototypical datasets [30]. Generally, metabolomics data preprocessing involves multiple computational steps with many parameters, which hampers reproducibility, scalability, and comparison across datasets. To address these challenges, Delabriere et al. developed SLAW (scalable LC-MS (liquid chromatography-mass spectrometry) analysis workflow), a metabolomics data preprocessing pipeline that includes an automatic parameter optimization procedure for peak-picking, alignment, and normalization across large metabolomics datasets [31].

After preprocessing, the next step in the metabolomics data analysis pipeline is compound identification. Identifying compounds and fragments in MS data is a difficult task, especially in untargeted MS data, usually done by comparing the measured accurate mass, retention time, and fragmentation pattern of a compound with metabolite and spectral databases (e.g. MetaboLights [32], Metabolomics Workbench [33], GNPS [34], METLIN [35], HMDB [36], and MiMeDB [37]). Combining several annotation sources has recently been shown to improve metabolite annotation in NMR datasets [38]. Meanwhile, a number of alternative approaches have recently been developed. One representative example is the work by Ross et al., who generated a large *in vitro* library of collision cross-section (CCS) values for drugs and drug metabolites and built a support vector regression model to predict them from structural information [39]. The CCS value is a unique physical property of a compound and thus a robust measurement across instruments; hence, it can be used to aid compound identification. Further, Zhou et al. compiled and curated a large CCS value atlas for ion mobility–MS from 14 different datasets and demonstrated an improved annotation performance for both known and unknown compounds [40]. Reder et al. developed Labeled Latent Dirichlet Allocation to map spectrum features to the chemical space of known structures as a supervised topic modeling approach, which allows for interpretable chemical structure prediction given tandem MS profiles [41]. Gao et al. took the approach of predicting molecular fingerprints directly from spectral information using convolutional NNs trained on spectra of more than 36,000 compounds from public databases [42]. MSNovelist predicts *de novo* compound structures from tandem mass spectra with recurrent NNs [43], while Retip offers a set of machine learning models to predict retention time of compounds, given an experimental training set collected by the user [44] (Figure 1).

Another challenge in compound identification arises from the fact that some spectral libraries are proprietary, while the publicly available ones are missing spectra for many compounds. One solution to overcome this challenge is to develop ways to generate *in silico* spectra. Competitve Fragmentation Modelling for Metabolite Identification (CFM-ID) is a package developed by Wishart lab [45] that utilizes machine learning to predict spectra given structural information about a compound. With its fourth iteration, CFM-ID has been benchmarked by Bremer et al. by comparing the predicted spectra with experimental ones found in the NIST20 database, who found that few predicted spectra have high similarity with their experimental counterparts [46]. The MS2Compound tool uses CFM-ID to generate a custom database with predicted spectra based on the user-defined compound list, which can subsequently be used for metabolite annotation [47].

Finally, after identifying metabolites present in a microbial sample, one needs to determine their reciprocal relationships with the microbes to gain mechanistic insights into metabolic interactions in microbial communities. On the one hand, some works try to predict microbial community diversity or composition from metabolite profiles [20,48]. For example, Han et al. used a random forest model to predict bacterial taxonomy given the metabolite profile trained on the in-house experimental dataset from 178 single microbes and 833 metabolites [20] (Figure 1). On the other hand, curated collections of microbiome–metabolome data allow to predict metabolite profiles given microbial composition [19,49,50]. Lu et al. use a Bayesian logistic regression model to predict metabolites of tryptophan given a taxonomic profile based on a curated database of 108 metabolites and 1334 human or mouse gut bacterial species [49]. Muller et al. generated a curated collection of paired fecal metabolome–microbiome data from 14 different cohorts (2900 samples from 1849 individuals) and demonstrated that a random forest model can predict abundance of >90 metabolites given microbiome composition data [19]. The tool MiMeNet takes advantage of NNs and paired metabolome–microbiome datasets to predict metabolite abundances and build microbe–metabolite interaction networks [50] (Figure 1). Le et al. proposed a sparse neural encoder–decoder network which not only predicts metabolite abundances from microbiome data but also allows to interpret microbe–metabolite links from the hidden layer of the network [51]. Given the dynamic nature of microbiome composition in the human gut, several packages were developed to specifically analyze time-series metabolomics data, such as MDITRE [52] and CGBayesNets [53]. Both of these tools combine Bayesian approaches with deep learning to predict human-interpretable rules for host status given taxonomic information. Taken together, approaches based on machine and deep learning pave the way forward in resolving compound identification and microbe–metabolite associations (Table 1), while their interpretability often remains challenging and could be enhanced in combination with knowledge-based approaches [23].

**Table 1:** Data-driven and knowledge-based tools for computational analysis of microbial metabolomics data that were developed within the past two years.

Tool (Language)	Goal	Input	Output	Principles
**Data-driven approaches**				
SLAW (Docker) [31]	Data preprocessing	MS spectra	A table of *m*/*z* values, retention times, and intensities	A containerized scalable and largely automated LC-MS processing workflow that includes automatic parameter optimization for data preprocessing steps (peak-picking, sample alignment, gap filling, MS2 extraction across samples).
MSNovelist (Docker) [43]	Compound identification	MS spectra	A table of structures in form of SMILES codes	Relies on SIRIUS and CSI:FingerID web service to predict molecular formulas from spectra. Then encoder–decoder recurrent neural network model is used to predict structures given the molecular formula.
Retip (R) [44]	Compound identification	Table of chemical descriptors (SMILES/InChI) with retention times as a training dataset	A ML model that can predict retention times	Various machine learning models, such as random forest, Bayesian-regularized neural network, XGBoost, light gradient-boosting machine and NNs, were trained to determine retention times from compound SMILES/InChi descriptors.
CFM-ID (Java) [45]	Mass spectra prediction	SMILES	Predicted spectra	All possible fragments are generated for a given molecule as a graph; the probabilities of each fragmentation are generated using machine learning.
MS2Compound (C#) [47]	Compound identification	List of compounds and descriptors (SMILES); MS spectra	Custom database of predicted spectra; compound identities from custom or built-in database	A GUI wrapper around the tool CFM-ID.
MiMeNet (Python) [50]	Integrative analysis of the paired microbiome–metabolome datasets	CSV files of metabolite and microbial count values	Microbe-metabolite interaction-score matrix	Trains a neural network, first by tuning the hyper-parameters, then performing a cross-validation to determine how well measured metabolites are predicted from microbial composition.
MB-SupCon (Python) [81]	Integration of microbiome and metabolomics data under a supervised contrastive learning scheme	16S rRNA amplicon sequencing data and metabolome data	Sample phenotypes (e.g. patient status)	A supervised contrastive learning model is trained to obtain the weights of the two encoder networks for microbiome and metabolome data. Microbiome encoder network is then applied to new microbiome data to obtain microbiome embeddings, which can then be used in a classifier to predict sample phenotypes.
CGBayesNets (MATLAB) [53]	Analysis of temporal variation of the gut microbiota	Microbiota abundance values	Sample phenotypes	Conditional Gaussian Bayesian networks that predict sample phenotypes (e.g. patient status) and provide information on feature importance for interpretation (e.g. biomarker discovery).
**Knowledge-based approaches**				
merlin (Java) [54]	Metabolic re-annotation and GSMM reconstruction	Bacterial genome files (fasta)	Genome-scale metabolic model (can be manually curated)	Genome-scale metabolic model reconstruction through genome annotation, reconstruction, gap filling, refinement and validation. Includes manual curation tools with GUI.
gapseq (R) [56]	Generation of GSMMs	Bacterial genome files (fasta)	Genome-scale metabolic model with gap-filled reactions based on databases and literature	Genome-scale metabolic model reconstruction pipeline that includes genome annotation, model reconstruction, gap-filling, refinement and validation.
metaGEM (Snakemake pipeline) [57]	Generation of GSMMs and prediction of metabolic interactions from metagenomic data	Bacterial genome or metagenome files (fasta/fastq)	Genome-scale metabolic models, list of metabolites exchanged in a given microbial community, donor microbe – metabolite – recipient microbe links	A pipeline that utilizes other tools such as CarveME, SMETANA, bwa, Prokka, Roary amongst others, in order to generate community metabolic models from metagenomics data, and predict metabolic interactions within these communities.
AMON (Python) [67]	Annotation of metabolite origins based on KEGG network	KEGG orthology identifiers and KEGG compound IDs	Compound-genome associations	Determines whether metabolites have been produced by the host or microbial communities by using KEGG orthology information to link compounds and host and bacterial sequences present in each sample.
MetOrigin (Web-based tool) [68]	Identification of metabolite origin based on seven knowledge databases	Metabolome and microbiome data	Metabolite-species associations (in the form of Sankey diagrams)	Integrates information from various databases to determine metabolite origin; performs pathway enrichment analysis and correlation analysis to perform microbe–metabolite network analysis.
MIMOSA2 (R) [63]	Metabolic model-based analysis of microbiome and metabolomics data	Microbiome data and metabolome data	Various tables describing relationships between taxa and metabolites	Constructs a metabolic model, then calculates metabolic potential scores for each taxon and metabolite based on the metabolic model. Given the metabolic potential scores, MIMOSA2 assesses whether they are significantly predictive of measured metabolite levels using a regression model.

## Knowledge-based approaches: metabolic networks and GSMMs

Metabolic networks and GSMMs are network-based approaches that use knowledge of metabolic pathways to analyze metabolomics data and model microbial communities. GSMMs are mathematical models which are reconstructed based on microbial genomes or metagenomes to incorporate metabolic reactions present in a cell [54, 55, 56, 57]. Once constructed, these models allow to generate hypotheses for the microbial system under study, such as which metabolites can be produced, consumed, or exchanged, and what metabolic interactions can happen between the community members [58]. Reiter et al. used GSMMs to predict which metabolites can be found in a microbial sample based on its genome and thus inform the development of an analytical method to screen its metabolome with MS, which they tested on yeast metabolism [59] (Figure 1).

After identifying the metabolites and microbial composition in the sample, associations between the microbiome and metabolome can be drawn using GSMMs, which has been demonstrated in several clinical applications [55,60,61] (Table 1). Hertel et al. used GSMMs to identify important reactions in the microbiome of colorectal cancer patients [62] (Figure 1). Proffitt et al. identify differences in specific metabolic pathways across metabolic disorders using GSMMs built based on metagenomic data [61], while Noecker et al. developed a package MIMOSA2 to predict the differences in metabolite abundances given metagenome composition using genomic and metabolic reference databases [63] (Figure 1). Further, Mujagic et al. used metabolic reaction network analysis to reveal the connection between stress and serotonin metabolism in irritable bowel syndrome [64]. Although useful to interpret and integrate metabolomics and metagenomics data and generate biological hypotheses, GSMMs suffer from the lack of standardization due to multiple incompatible databases that are used to construct the models and non-unified nomenclature [65]. Another issue with GSMMs is the uncertainty of the construction of the model due to the knowledge gaps, which can be partially overcome through probabilistic approaches and ensemble modeling [29].

Furthermore, databases of biochemical reactions can be used to connect microbiome and metabolome without the use of GSMMs [66, 67, 68] (Table 1). Levi et al. used the Kyoto Encyclopedia of Genes and Genomes (KEGG) database [69] to determine functions of microbes in communities [66], Shaffer et al. developed a KEGG-based metabolic network analysis tool to separate host and microbial metabolites [67] (Figure 1), while Yu et al. developed a web application to scour multiple metabolite databases to determine the origin of metabolites for a given metabolomics sample [68]. In addition to generic databases such as KEGG, more organism- or environment-specific databases that connect microbiome and metabolome and provide experimental datasets become available, such as MiMeDB [37], gutSMASH [70], and paired omics data platform [71]. Overall, while being limited by the existing database knowledge, metabolic networks and GSMMs provide a more tangible and explainable method to identify metabolites and separate microbial contributions to metabolites in mixed samples compared to the black-box approach of machine learning.

## Integrating data-driven and knowledge-based approaches

Both data-driven and knowledge-based approaches can be combined to improve one another. Sen et al. gave an overview of the recent applications of deep learning to GSMMs, such as using machine learning to fine-tune reaction constraints, gap-fill missing reactions in automatically reconstructed models, or pick the model parameters [23]. Moreover, random forest classifiers combined with GSMMs have been used to guide experimental efforts by predicting which data are more informative to reduce the knowledge gaps between GSMMs simulations and observed experimental phenotypes [72].

Knowledge-based approaches combined with data-driven approaches, in turn, can improve interpretability of the latter [73]. Hertel et al. used a more general data-driven statistical approach to identify differentially abundant metabolites and species in fecal samples of colorectal cancer patients and integrated it with GSMMs to mechanistically link altered glutarate levels to lysine fermentation by *Fusobacterium* species [62]. Another study developed a pipeline that uses kernel regression to link genomics and metabolomics data given machine learning-based predictions of metabolic functions, metabolomics databases, and paired metabolomics and genomics datasets [74]. Finally, computational metabolomics can be enriched by methods traditionally used in phylogenetic analysis: Tripathi et al. used tree-guided data exploration tools to aid in compound identification in MS data represented as hierarchically organized molecular fingerprints [75].

## Conclusions, challenges, and future directions

The field of microbial metabolomics faces many challenges — from data preprocessing and compound identification and annotation, to disentangling metabolite origins from mixed microbial samples. Data-driven and knowledge-based approaches offer a plethora of methods to tackle these problems, and new methods are being actively developed. Knowledge-based approaches, such as metabolic networks and GSMMs, can be used to predict metabolites present in a sample and generate hypotheses, while data-driven approaches, such as machine/deep learning, can aid in compound identification through prediction of compound spectra and other properties, or detecting microbe–metabolite relationships in large-scale datasets. While many application examples mentioned in this review focus on bacterial metabolomics, most of the overviewed methods are general and can be applied to fungal metabolomics datasets, recently reviewed by Shankar and Sharma [76]. Integrating the two types of approaches offers even more potential solutions to the challenges in this field. In the near future, other types of methods, such as graph NNs [77], may become more widespread to investigate microbial metabolism due to the graph-based nature of microbial metabolomics datasets and microbe–metabolite associations.

Both data- and knowledge-based approaches for microbial metabolomics data annotation and interpretation rely on the quality and the availability of the underlying data and knowledge and thus are hampered by the incompleteness of spectral databases and microbial genome annotation, small dataset sizes, lack of data standardization, and proprietary databases [15,18,23,73,78]. Much remains to be improved in data and processing standardization, and ensuring open access to databases and raw data repositories is crucial to assist in reporting and increasing the annotation confidence level [79,80]. To advance the analysis of primary microbial metabolism, we need a community effort to produce and openly share publicly available, curated, and annotated single and paired microbial metabolomics datasets, both from microbial communities and single microbial species [14,19,23].

Box **Machine learning** is a branch of computer science that uses algorithms to create models that can learn the relationships between input and output variables from data by adjusting parameters based on a defined cost function.

**Deep learning** is an umbrella term for the use of models including any neural network (NN) with many layers, including, among others, convolutional neural networks (CNNs) and recurrent neural networks (RNNs). NNs are a class of models in machine learning, for which computational architectures are defined as simple processing units (artificial neurons) organized in graph-based topologies. NNs are typically composed of connected layers each containing several neurons, where each neuron takes a weighted sum of inputs to which a nonlinear activation function is applied. There are different types of NNs that are in use currently, mostly differing on the topologies (architectures) and types of layers used.

**Convolutional NNs** are a type of NNs where at least one of the layers in the NN is convolutional, typically also including pooling layers. The convolutional layers apply filters to the input data to extract relevant features, while the pooling layers reduce the dimensionality of the input. CNNs are most commonly used on image or other multidimensional data. Recurrent NNs, on the other hand, are often used in sequence-based inputs (e.g. text processing or biological sequences) and can allow output from some nodes to affect subsequent input to the same nodes.

**Neural encoder–decoder networks** are special cases of NNs, which can be trained to encode/decode different raw data (e.g. text, images, omics data) into more compact numerical vector representations. These might be used for feature generation (encoders) and for generative models (decoders), among other applications.

**Support vector regression** is a regression method that aims to minimize the amount by which the predicted values, given by a hyperplane, deviate from a fixed margin around the actual values. This margin is defined by two parameters: epsilon and C. Epsilon determines the width of the margin, while C controls the trade-off between maximizing the margin and minimizing the error.

**Bayesian logistic regression** is a classification algorithm that aims to predict binary dependent variables given one or more independent variables. The probability of the dependent variable taking a certain value is modeled as a function of the independent variables using a logistic function. The model also includes prior distributions on the coefficients of the independent variables, which capture any prior knowledge or beliefs about the values of the coefficients before seeing the data.

**Random forests** are a class of machine learning algorithms used for classification or regression that are based on an ensemble of decision trees. A decision tree is a tree-like model where nodes represent decision rules based on the input feature values, branches correspond to different outcomes of the decision rules applied to the features, and leaves represent the final labels.

**Labeled Latent Dirichlet Allocation** is a machine learning model in natural language processing, specifically in topic modeling, that determines labels of the observations (e.g. words in a document) and thus assigns each observation to a specific topic or group.

**NIST20** is a database of tandem mass spectra collected by the U.S. National Institute of Standards and Technology (NIST). NIST databases are released periodically with an increasing collection of spectra, with NIST20 being the most recent version to date.

**Metagenome** is the collection of genome sequences recovered from genetic material extracted from a mixed sample. Metagenomics is the study of metagenomes; by analogy, metatranscriptomics and metaproteomics are studies of collective transcriptome and proteome material recovered from mixed samples, correspondingly. Metagenomics, metatranscriptomics, metaproteomics, and metabolomics are often collectively referred to as omics.

## Funding

This work was funded by the European Molecular Biology Laboratory. MZ-K acknowledges support from the 10.13039/501100001961AXA Research Fund.

## Declaration of competing interest

The authors declare that they have no known competing financial interests or personal relationships that could have appeared to influence the work reported in this paper.

## Data Availability

No data was used for the research described in the article.
